# Student engagement during emergency remote teaching: A scoping review

**DOI:** 10.1186/s40561-023-00240-2

**Published:** 2023-03-14

**Authors:** Dong Yang, Huanhuan Wang, Ahmed Hosny Saleh Metwally, Ronghuai Huang

**Affiliations:** 1 Smart Learning Institute, Beijing Normal University, Beijing, China; 2 National Engineering Research Center of Cyberlearning and Intelligent Technology (China), Faculty of Education, Beijing Normal University, Beijing, China; 3 Education Technology Department, Faculty of Education, Helwan University, Cairo, Egypt

**Keywords:** Student engagement, Covid-19, Scoping review, Emergency remote teaching, Teaching strategy

## Abstract

Research on student engagement has recently gained popularity as it can address problems such as early dropout and poor achievement. The growing interest in investigating student engagement during the Covid-19 pandemic is reflected in increased publications addressing this topic. However, no review provided research evidence and an overview of existing literature on student engagement during emergency remote teaching (ERT). We reviewed how student engagement studies were undertaken during ERT from three perspectives: (1) the landscape of studies, (2) methodologies issues, and (3) the strategies used to facilitate student engagement. 42 articles were analysed from an initial pool of 436 search results. The findings illustrate that current studies were predominately undertaken in the United States (36%) and China (22%) with focusing on STEM subjects as a dominant discipline. The literature was largely inconsistent in defining and measuring student engagement. In addition, the majority of studies (57%) investigated students’ engagement from the perspective of students, unlike other stakeholders. The most prominent finding is that ERT promoted several important engagement strategies, including motivational factors, teachers’ facilitation, a hybrid learning model, and using learning technologies to boost students’ engagement.

## Introduction

Students’ engagement in learning has gained increasing attention recently. It was defined as “the time and energy students devote to educationally sound activities inside and outside of the classroom and the policies and practices that institutions use to induce students to take part in these activities.” (Kuh, 2009, p.7). Student engagement was also described as positive emotions, learning strategies (Lau & Roeser, 2002), and institutional efforts to enrich students’ learning experiences and performance (Trowler, 2010). Despite the large variation in how engagement has been defined, there is some consensus that engagement is a multi-faceted construct that unites varying forms of engagement (Fredricks et al., 2016).

Due to the Covid-19, school teaching and learning were disrupted and forced to be delivered online. As of 21st June 2021, there are still 16,692,641 learners being excluded from the normal learning process (UNESCO, 2021). Since student engagement is associated with academic achievement (Appleton et al., 2008) and mental health (Steele & Fullagar, 2009), the Covid-19 pandemic may put student engagement at risk. The damage of pandemic to individuals goes from learning loss to even loss of earnings in students’ future working life (Dorn et al., 2020b). Such a sudden shift makes educationalists and other stakeholders deeply concerned about engaging students in times of crisis. As a result, various efforts were made to “maintain” or “facilitate” student engagement (e.g., Chiu, 2021; Zhang et al., 2021).

The current state of learning engagement has been addressed in some reviews (i.e., Bond, 2021; Harbour et al., 2015; Schindler et al., 2017). However, recognizing a broad overview of the state-of-the-art and exploring the best practices that facilitate student engagement during the pandemic is still undetermined. Thus, this review aims to provide a comprehensive summary of the research trends and pillars of students’ engagement during the Covid-19 pandemic. We believe that this review is significant and carries potential contributions. First, since the impact of the Covid-19 on education may last a few more years (Schleicher, 2020), a summary of current evidence can provide insights for educators and researchers regarding students’ engagement. Second, it can advance the knowledge for fostering students' engagement in a time of crisis, which could help to overcome learning engagement challenges in the future.

To fill the knowledge gap regarding facilitating student engagement in the global pandemic, our scoping review seeks to answer the following questions:

- What are the landscapes (i.e., country, participants' profile, educational settings, academic cooperation, and conceptualization) of students’ engagement research?
- What are the characteristics of methodology (i.e., types of data, approaches, instruments in use) used in the published studies?
- What are the adopted strategies to foster student engagement during the Covid-19?

## Methods

We conducted a scoping review of the published literature on empirical studies of student engagement during the Covid-19 pandemic. Scoping review is useful for examining emerging evidence when it is still unclear (Armstrong et al., 2011) and mapping the evidence base (Salmela-Aro et al., 2021). It is appropriate for clarifying key concepts/definitions in the literature, examining how research is conducted on a certain topic, identifying key factors related to a concept, and identifying and analyzing knowledge gaps (Munn et al., 2018). Therefore, we opt for a scoping review method to understand how studies on student engagement were conducted during the Covid-19 pandemic, focusing attention on the adopted strategies to promote student engagement. We followed the Preferred Reporting Items for Systematic reviews and Meta-Analyses extension for Scoping Reviews (PRISMA-ScR; Tricco et al., 2018) framework when conducting our scoping review.

### Searching strategy

The literature search was performed within the following databases: Academic Search Complete, Emerald Journals, ERIC, ISI Web of Knowledge, JSTOR, PsycINFO, Science Direct, and Wiley Online Library. Those databases were chosen for their breadth in education, psychology, technology, and social science. We included peer-reviewed journal articles published from 2020 to August 2021; the time Covid-19 began until the date of searching databases.

Three key search terms used on the databases were: “student engagement,” “Covid-19,” and “facilitate.” Similar terms such as “involvement” and “participation” can be found in the literature. However, we chose to focus only on articles using the word “engagement” in the abstract section, expecting that it would have direct connections with student engagement. We used alternative terms in the search strings regarding engagement to expand the results, as described in Table 1.

**Table 1:** Search terms and strings

Items	Search terms	Boolean
Student engagement	“School engagement” OR “engagement in school” OR “student engagement” OR “pupil engagement” OR “learner engagement” OR “emotional engagement” OR “cognitive engagement” OR “behavioural engagement” OR “agentic engagement” OR “academic engagement”	AND
Covid-19	“COVID-19” OR “Covid19” Covid-19 pandemic” OR “ARS-CoV-2″ OR “novel coronavirus” OR “emergency remote teaching” OR “time of transition” OR “time of change” OR “time of disruption”	AND
Facilitate	“Facilitate” OR “foster” OR “boost” OR “promote” OR “nurture” OR “cultivate” OR “enhance” OR “strengthen” OR “sustain” OR “maintain” OR “predict” OR “impact” OR “affect”	

### Inclusion and exclusion criteria

**Fig. 1:**
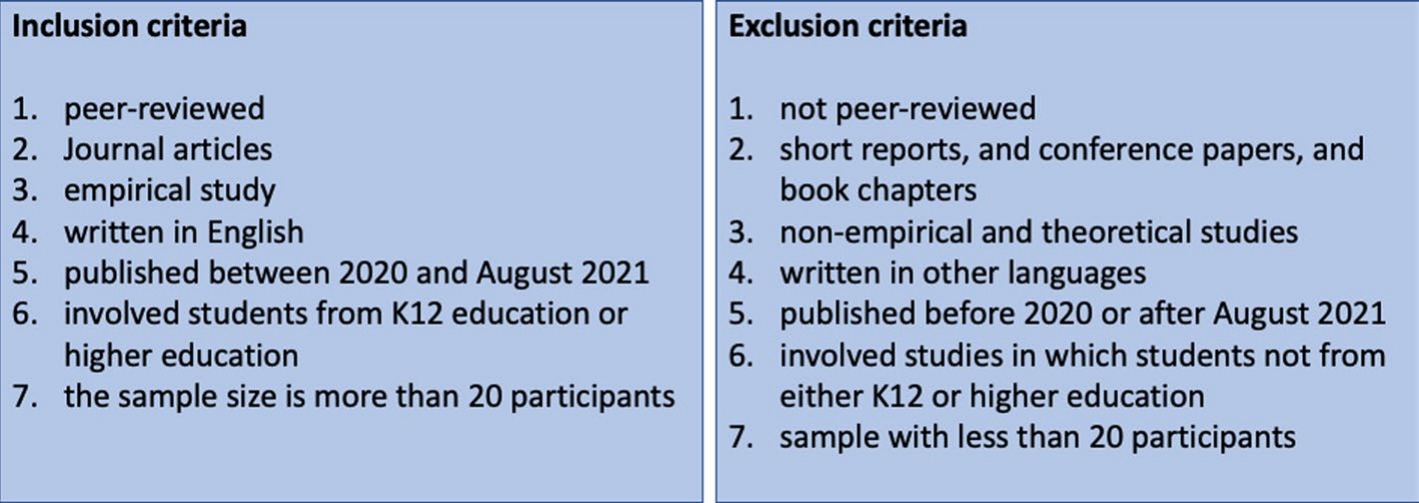
Inclusion and exclusion criteria

To ensure a quality collection of literature, we only chose peer-reviewed journal articles published in English. Since one objective is to explore the adopted strategies to facilitate student engagement, we only selected the empirical studies. Reviews, short reports, and conference papers were also excluded, as shown in Fig. 1. Moreover, we decided to keep only studies with a sample size of at least 20 as suggested by Miller (1991).

### Screening process

**Fig. 2:**
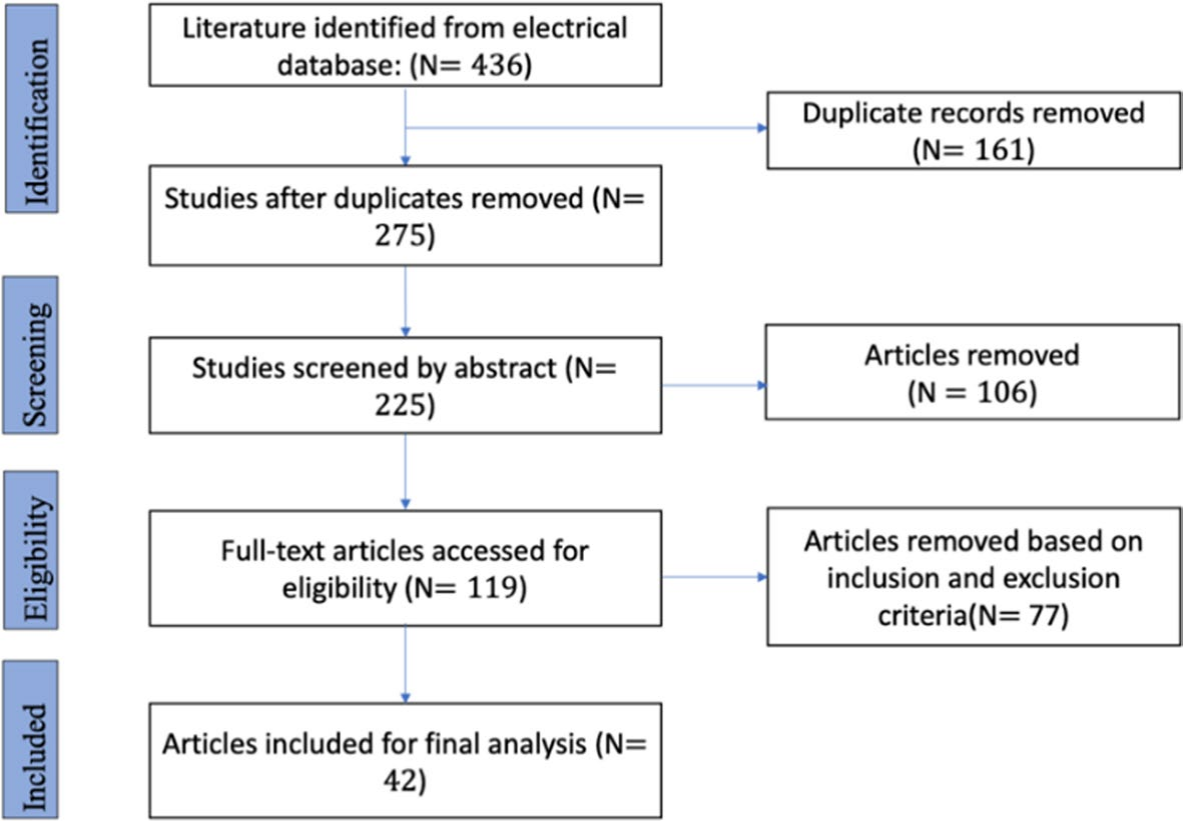
Literature screening process

Database literature search generated 436 records. Next, we deleted a total of 161 duplicate records. Then, we reviewed all abstracts for relevant information on the Covid-19 pandemic and students' engagement. As a result, 106 unrelated records were removed. To control the quality of the included studies, we only included the articles with a sample of more than 20 participants. This analysis was performed by reading the full text and applying the inclusion criteria. Accordingly, our final review pool was narrowed down to 42 articles. The screening process is shown in Fig. 2.

### Data analysis

Before analysis, all researchers agreed on a coding schema, including bibliometrics of the publication, countries where studies were carried out, the educational setting of the study, participants' profiles, the subject area where student engagement was researched, and strategies adopted to foster student engagement. The detailed coding schema can be found in Appendix A. Following the coding schema, all authors analyzed the literature collaboratively. Two researchers reviewed each article and coded them to identify the landscape of publications, the conceptualization of engagement, methodology issues, and strategies used to facilitate student engagement. During the analysis, agreement and shared understandings of the coding, categorization, and themes were reached via multiple analyses and dialogue between researchers.

## Findings

### What are the landscapes of the identified studies?

#### Countries

We identified countries by the affiliation of the first author. Overall, most of the studies (88%) were conducted in North America (N = 19; 45%) and Asia (N = 18; 43%). Among North America, the United States lead 15 studies (36%) occurred, and Canada led four studies, accounting for around 10% of the total amount. Meanwhile, China (N = 9; 22%) was the top Asian country in the number of studies. The rest of the studies conducted in Asia (N = 9) identified were scattered across India and Japan. Surprisingly, we found only two publications from Europe countries, representing about 5% of the total amount. This is the same situation in South American countries, where only two studies were published. See Table 2 for the full list of included studies.

**Table 2:** A summary of countries and participants of engagement research

Country/Place of study	n	Article	Participants
Australia	1	1. Ewing and Cooper (2021)	15 K-12 students, 12 parents and 13 teachers
Brazil	1	1. Lima et al., (2020	50 undergraduate students
Canada	4	1. Walker and Koralesky (2021)	145 undergraduate students & 13 university teachers
		2. Daniels et al. (2021)	98 university students
		3. Code et al. (2020) 4. Petillion and McNeil (2020)	42 secondary specialist TE teachers 71 undergraduate students
China	9	1. Chiu (2021) 2. Jia et al. (2020) 3. Yang et al. (2020) 4. Zhang et al. (2020) 5. Chiu (2021) 6. Xu et al. (2020) 7. Zhao et al. (2021) 8. X. Yang et al. (2021) 9. Chan et al. (2021)	1201 grade 8–9 students 47 undergraduate students 357 grade 3–5 students 1119 undergraduate & graduate students 36 secondary school students & 18 secondary school teachers 46 undergraduate students 1040 undergraduate students 377 undergraduate students 56 undergraduate students
Germany	1	1. Daumiller et al. (2021)	80 faculty members
India	2	1. Deka (2021) 2. Tigaa and Sonawane (2020)	290 both undergraduate & graduate students 150 undergraduate students
Ireland	1	1. Bray et al. (2021)	723 secondary school teachers
Japan	1	1. Abou-Khalil et al. (2021)	313 both undergraduate and graduate students
Korea	1	1. Lim et al. (2021)	291 undergraduate students
Mexican	1	1. Roque-Hernández et al. (2021)	1417 undergraduate students
Palestine	1	1. Khlaif et al. (2021)	34 middle school students
Pakistan	1	1. Shah et al. (2021)	689 undergraduate students
Philippines	1	1. Lapitan et al. (2021)	59 undergraduate students
Saudi Arabia	1	1. Al-Bogami and Elyas (2020)	20 middle school students
Turkey	1	1. Kurt et al. (2021)	20 K-12 students & 22 teachers
USA	15	1. Chu (2020) 2. Nickerson and Shea (2020) 3. Perets et al. (2020) 4. Alpert et al. (2021) 5. Wester et al. (2021) 6. Orlowski et al. (2021) 7. Senn and Wessner (2021) 8. Erickson and Wattiaux (2021) 9. Orlov et al. (2021) 10. Ranga (2020) 11. Baldock et al. (2021) 12. Mejia (2020) 14. Davidson et al. (2021) 15. Krause and Moore (2021)	107 undergraduate students 113 undergraduate students 30 undergraduate students & 2 university teachers & 2 peer tutors 68 undergraduate students 73 undergraduate students 156 undergraduate students 32 undergraduate students 261 undergraduate and graduate students & 10 instructors 809 undergraduate students 111 undergraduate students 152 undergraduate students 70 undergraduate students 10,130 middle school students 68 undergraduate students & 34 mentors

#### Samples and participants

The reviewed studies had been conducted on a wide range of samples, ranging from a small group of students to large-scale studies involving thousands of students. The sample of the 42 studies comprised 21,109 participants. The average number of participants is around 503. Out of the 42 studies, 45% (N = 19) included a sample of fewer than 100 participants, while 35% (N = 15) used samples ranging from 100 to 500 participants. Only eight (20%) studies had samples with more than 500 participants. See the full list in Table 3.

**Table 3:** The Level of students studied

Grade	No. of the studies	Percent (%)
Grades K-6	2	4.8
Grades 7–12	4	9.5
Both K-6 and 7–12	4	9.5
Undergraduate students	26	61.9
Graduate students	0	0.0
Both undergraduate and graduate	4	9.5
Unknown	2	4.8
Total	42	100

#### Educational settings

The analysis of the educational settings combines grade and subject areas. We found that 62% (N = 26) of the studies recruited undergraduate students as participants, while 24% (N = 10) of the studies involved participants from the K-12 level. In addition, four identified articles (9.5%) contained both undergraduates and graduates as participants, and two studies did not report the education level of the participant population (See Table 3). Thus, studies on student engagement were mostly focused on higher education.

Many identified studies did not state the subject (N = 12; 26%). Besides, approximately one out of three studies (N = 18; 38%) is located in the field of STEM (Science, Technology, Engineering, & Mathematics). The rest of the studies are scattered across Medicine and Nursing, Economics and Business, Education, and Psychology as tabulated in Table 4.

**Table 4:** Summary of subject areas addressed in the literature

Subject/disciplinary	No. of the studies	Percent (%)
STEM	18	38
Medicine & Nursing	7	15
Economics & Business	6	13
Education	2	4
Psychology	2	4
Uncertain	12	26
Total	47*	100

^*^In several studies, samples feature multiple subjects

#### Research cooperation

The research cooperation has been identified through reviewing the authorship and institutions’ data. Overall, 12 (28%) articles as identified were single-authored or conducted by two researchers within an institution. We treated a publication with three or more authors as multi-authored. In this review, half of the studies (N = 21; 50%) were multi-authorship, at a national level. Most often, they came from one single institution (N = 13; 31%). International cooperation appeared in research nearly less than a quarter of the total number in the aggregation (N = 9; 21%). In addition, the majority (N = 8; 19%) of such publications were multi-authored and involved two or three academic institutions. This finding denotes that there is insufficient international cooperation in learning engagement research during the pandemic, which is unexpected in the context of a global pandemic.

#### Conceptualization

In terms of conceptualization, eight studies (19.2%) conceptualized student engagement using *the three-dimensional definition* proposed by Fredricks et al., (2016a, 2016b), and eight studies (19.2%) referred to *the theory of Community of Inquiry* as developed by Anderson and his colleagues (2008). Three studies (7%) conceptualized engagement as integrating both behavioral and affective aspects of engagement, while two studies (4.8%) used the theory as suggested by Reeve and Tseng (2011), which added agentive engagement on top of the three-dimensional framework proposed by Fredricks and his colleagues (2004). Besides, many articles (N = 19; 45%) failed to provide conceptual bases for student engagement, suggesting a poor theoretical grounding. Details of conceptualization can be seen in Table 5.

**Table 5:** Theoretical perspectives of conceptualizing engagement

Dimensions/theories	No. of the studies (%)	Examples
Community of INQUIRY (CoI)	8 (19.2)	Jia et al. (2021), Kurt et al. (2021)
Behavioural/affective/cognitive	8 (19.2)	Wester et al. (2021); Orlowski et al. (2021)
Behavioral/affective	3 (7)	Al-Bogami and Elyas (2020), Bray et al. (2021)
Behavioural/affective/cognitive/agentic	2 (4.8)	Lima et al. (2020), Yang et al. (2020)
Affective/cognitive	1 (2.4)	Walker and Koralesky (2021)
Self-determination theory (SDT)	1 (2.4)	Shah et al. (2021)
Others	19 (45)	Davidson et al. (2021); Domina et al. (2021)

### What were the characteristics of methodology among the identified studies?

Regarding the methods, survey is the dominating method used to investigate student engagement during the crisis (N = 24; 57%). A semi-structured interview was also used solely in several studies (N = 5; 12%). Besides, mixed methods that integrate various data forms were popular among the studies (N = 11; 26%). For instance, five studies (12%) used both questionnaires and semi-structured interviews; three studies (7%) applied both questionnaire and trace data. Table 6 shows the full list of types of data.

**Table 6:** Types of data used for studying student engagement

Data collected	No. of the studies	Percent (%)
Questionnaire data	24	57.0
Interview data	5	12.0
Questionnaire data & interview data	5	12.0
Questionnaire data & trace data	3	7.0
Trace data*	2	4.8
Interview data & observation data	1	2.4
Questionnaire data & observation data	1	2.4
Questionnaire data & interview data & trace data	1	2.4
Total	42	100

*Trace data refers to records of activity undertaken through learning management systems

Surprisingly, most of the identified articles relied on an incomplete report regarding the specific instrument used to measure student engagement (N = 29; 69%). For the remaining studies (N = 13; 31%), four studies applied Dixson’s Online Student Engagement Scale (Dixson, 2015), and three studies used the behavioral, emotional, and cognitive engagement scale developed by Sun and Rueda (2012). Besides, two studies utilized Skinner’s Engagement Questionnaire (Skinner, 2009). Other studies used instruments such as the Utrecht Work Engagement Scale (N = 1; 2%) (Schaufeli et al., 2006), Community of Inquiry (CoI) framework scale (N = 1; 2%) (Arbaugh et al., 2008), and engagement scale developed by The National Centre for School Engagement (N = 1; 2%) (Finlay, 2006).

### What are the adopted strategies to foster student engagement during the Covid-19?

The strategies used to promote student engagement were summarized in Table 7. We found that 17 (40%) studies discussed the substantial role of psychological factors in promoting student engagement during the Covid-19 pandemic. Chiu (2021) showed the effectiveness of applying self-determination theory (SDT) to facilitate student engagement in online learning. They found that the realization of online learners’ need for autonomy, competence, and relatedness can facilitate online learning engagement. Chu (2020) investigated how positive psychology teaching strategies can benefit student engagement. In addition, increasing students’ self-efficacy (Yang et al., 2021); setting proper achievement goals (Daumiller et al., 2021) and enough social presence (Orlowski et al., 2021) have been reported as effective strategies to promote student engagement.

**Table 7:** Summary of strategies used to facilitate student engagement during the Covid-19

Category	Strategies (example)	No. of the studies (percent)
Psychological & motivational factors	Positive psychology (i.e., growth mindset) (Chu, 2020); self-efficacy (Yang et al., 2020); adaptability (Zhang et al., 2020); achievement goals (Daumiller et al., 2021); Social presence (Orlowski et al., 2021); perceived psychological needs (Chiu., 2021)	17 (36%)
Flexible pedagogy	Online peer-peer mentoring programme (Krause & Moore, 2021); Synchronous and asynchronous learning (Petillion & McNeil, 2020; Ranga, 2020); student & instructor chrematistics, course design/content., learning environment (Deka, 2021); student-content interaction (screen sharing, summaries, and class recordings) (Abou-Khalil et al., 2021)	11 (25%)
Teacher’s facilitation	Teachers’ presence & quality of content (Khlaif et al., 2021); teacher's ability (to support hands-on competency dev.) (Code et al., 2020); teacher facilitation (Using wechat tool) (Xu et al., 2020); global digital social learning as teaching stragety (Davidson, 2021)	8 (18%)
Technologies & digital tools	Use of digital tools such as Zoom and Slack (Nickerson et al., 2020); VR technology(Alpert et al., 2021), iPad Apps (Al-Bogami & Elyas, 2020)	6 (16%)
Others (i.e., infrastructure)	Access to high-speed Internet and Internet-enabled or electricity-enabled devices (Domina et al., 2021; Tigaa & Sonawane, 2020)	2 (5%)
Total		44*

*In four studies, at least two categories of strategies were applied simultaneously

Approximately, one out of four studies (N = 11) discussed how flexible pedagogy is related to better engagement. Several studies (Petillion & McNeil, 2020; Ranga, 2020; Walker & Koralesky, 2021) investigated how the use of synchronous or/and asynchronous learning holds the potential in promoting student engagement in ERL sessions. Krause and Moore (2021) found that applying for an online peer mentoring program in times of crisis was effective in promoting undergraduates’ online learning engagement and satisfaction. In addition, studies also discussed how fostering interaction holds promise to facilitate student engagement in (mostly) remote learning situations. For instance, strategies to boost learner-content interaction using screen sharing, making more course summaries, and recording classes were positively correlated with student engagement (Abou-Khalil et al., 2021). Strategies to promote peer-peer and student–teacher interactions using flipped course format (Jia et al., 2020) are auspicious strategies to enhance student engagement. Therefore, flexible pedagogy seems to be a salient factor that facilitates student engagement.

Teacher’s facilitation appears to be another key antecedent of student engagement reflected in the reviewed studies (N = 8; 18%). The literature emphasized two major forms of teacher facilitation. The first one is teachers’ support for students. For example, a teacher's presence in online courses is significant for scaffolding and reducing distance in learning to facilitate learning engagement (Abou-Khalil et al., 2021). Teachers’ hands-on support for student competency development is influential to students’ learning motivation and engagement (Code et al., 2020). Since face-to-face connection is limited because of school closure, teachers’ role in enhancing the meaningful connection with students is prominent (Bray et al., 2021; Lima et al., 2020). The second form concerns teachers’ use of technology. Several studies examined how teachers used digital tools and platforms to facilitate engagement (e.g., Al-Bogami & Elyas, 2020; Davidson et al., 2021; Xu et al., 2020). Nickerson and Shea (2020) figured out that the proper use of digital tools is substantial for engagement, while Alpert et al.(2021) examined the value of a new technology-based approach, VRO (Virtual read-out), in remote clinical radiology education programs for enhancing engagement. To wrap it up, the combination of teachers’ pedagogical competency and appropriate use of technologies holds the potential to promote student engagement during ERL.

The adopted strategies sought to respond to several challenges of students’ engagement during the Covid pandemic. The emerging challenges can be summarised as technical challenges; student challenges; and teaching challenges (Fhloinn & Fitzmaurice, 2021). These challenges included interaction limitations with students and teachers, lack of consistency in the types of courses, and taking part in long synchronous lessons (Stewart & Lowenthal, 2022). Moreover, Tulskar and Turunen (2022) figured out other challenges, such as distractions at home, communication with classmates and teachers, social isolation, and technical issues. Therefore, educational institutions should be aware and ready to face such challenges when shifting the pedagogical practices during emergency remote teaching.

## Discussion

This review explored the basic pillars and landscapes of student engagement during the Covid-19 pandemic. Using a scoping review approach, we found the available evidence as presented below:

First, in terms of landscapes, most of the studies were conducted in the United States. China occupied second place in the rank. Student engagement studies during the Covid-19 period seem to be underrepresented across European and African countries. Future studies are needed to explore the current practices in these countries. Furthermore, while some studies featured international cooperation efforts, research cooperation (both at the national and international level) is still limited, as plenty of the identified articles were single-authored or conducted by two authors within one institute. The reviewed studies did investigate student engagement across multiple subjects and different levels of education, with STEM subjects and college students as the most common highlights. However, we suggest that more attention should be paid to K-12 education, as it tends to be one of the most affected groups during the Covid-19 (Dorn et al., 2020a).

Second, it seems that still, no consensus existed on the *definition* and *measurement* of student engagement among scholars (Appleton et al., 2008; Bond et al., 2020). In our study, most of their definitions tend to explain it from the three dimensions framework as proposed by Fredricks et al. (2004). This also corresponded to how student engagement was measured across the identified studies. Similar trends were also identified in recent reviews (Yang et al., 2018; Salmela-Aro et al., 2021). As Boekaerts (2016) stated, it is salient for each research project to begin with a clear definition of their understanding. In addition, to measure student engagement, most of the identified studies used the engagement scale developed by Sun and Rueda (2012). Such a three-dimension framework seems to be predominantly used in both conceptualization and measurement of student engagement during the Covid-19 pandemic. Future research should reach a standard and a common understanding in terms of engagement’s definition and measurement. This consensus will clarify and differentiate engagement from other constructs, which facilitate recognizing the relations between context, engagement, and adjustment (Fredricks et al., 2016).

Third, methodology. We found most of the studies used either questionnaire data or interview data. This is followed by mixed methods combining questionnaire and interview data, or questionnaire and trace data. Thus, using questionnaires and semi-structured interviews is still dominating. Other methods, such as observations or behavior tracking were rarely used. Although self-report surveys have the merits of being practical, cost-effective, and easy to administer in classroom settings (Fredricks & McColskey, 2012), they are also useful for measuring affective and cognitive engagement (Appleton et al., 2008). However, students may not accurately respond under some conditions. As a result, self-reports may not reflect their actual behaviors or strategy use (Appleton et al., 2006). We suggest examining students’ engagement behaviors rigorously by using observation or trace methods that can avoid students’ subjective biases in self-reporting (Salmela-Aro et al., 2021) and employing mixed methods for future studies.

Fourth, for facilitating strategies, the findings covered that psychological (especially motivational) factors, teachers’ facilitation, and the use of technology and digital tools play effective roles in boosting engagement during the crisis. Specifically, we found motivational factors such as self-efficacy, goal settings, positive psychology, and flexible instructional approaches such as applying a hybrid learning model, and integration of synchronous and asynchronous learning were predominant in fostering engagement. Teachers’ role as facilitators of student engagement has been proved in previous studies. For example, teachers’ beliefs and behaviors (van Uden et al., 2013, 2014); autonomy (Lietaert et al., 2015); facilitation in an online learning environment (Xu et al., 2020). However, we also noticed that during emergency remote teaching period, some less developed regions have problems with infrastructure such as high speed Internet and learning equipment (i.e., Domina et al., 2021). In our review, limited studies concerned the student engagement of vulnerable groups during pandemic, this is an opportunity for future studies.

At times of Covid-19, a sudden transition to remote learning may lead to anxiety, stress, or even dropout among students (Dorn et al., 2020b), thus, strategies for enhancing engagement should be emphasized and worth further investigation. Code and colleagues (2020) argued that the Covid-19 has shifted the traditional pedagogy into a pandemic-transferred one and that teachers’ traditional curriculum-prescribed competencies are problematic in crisis. As a result, student motivation and engagement were affected. Therefore, teachers should know what technologies/tools students will be using, and choose appropriate approaches when delivering courses. Hence, we suggest organizing a tailor-up professional development program. In the training program, proper use of digital tools and technologies (i.e., AR to boost online interaction), positive psychology, or flexible pedagogy should be prioritized.

## Conclusion

Student engagement during the Covid-19 pandemic was explored in a scoping review. The main takeaway is that more diversity is required in further studies, including more research output from European and African countries, involving different educational stakeholders such as parents/guardians, principals, and teachers, applying mixed-methods approaches, and paying more attention to vulnerable student groups.

Based on our analysis, firstly, there is a need to have more research outputs on the topic of student engagement during pandemics in both European and African countries. In this disrupted educational world, it is salient to share good practices and experiences to facilitate student engagement with each other across the globe. Secondly, to promote global cooperation, consensus should be gained among scholars in defining and measuring engagement. Third, the potential research could apply mixed-methods, using multimodal data sources and involving opinions from different stakeholders. During the pandemic, students have to suddenly transfer to emergency remote teaching, except for their ability to adapt to the new normal, firm support from families, schools, teachers, or even enterprises is salient. This requires us to investigate engagement via different stakeholders, to explore how the joint efforts could facilitate student engagement in the “new normal.” Furthermore, the role of technology, especially how AI and emerging technologies can enhance students’ learning and social interaction, should be emphasized. This requires well-empower teachers with TPACK knowledge and firm beliefs to motivate student in “troubled waters. To that end, when needed, an individualized teacher training program to train teachers better choose and handle digital content and tools, should be conducted. We hope that this scoping review will provide a base for what needs to be done in the foreseeable future.

## Limitations

Several limitations should be acknowledged. First, using a scoping review, we focused on how research on student engagement was conducted during the Covid-19 pandemic. We only searched studies that were conducted from 2020–August 2021. However, the pandemic lasted longer than we expected. Thus, we failed to include articles published afterwards, or we cannot access how the dynamics of student engagement differ across various contexts, such as educational levels and stages of Covid-19, due to limited literature and relevant short time frame. Second, this review has the “file drawer problem.” Our scoping review included 42 studies. We may still ignore some studies despite using the most relevant search terms. According to Dalton et al. (2012), the such problem does not generate inflation or threaten literature review results. Future studies can include more publications and examine how those dynamics changed during the pandemic using systematic literature review and meta-analysis. Those can be interesting and crucial topics to explore, considering that the damage of the pandemic to individuals goes from learning loss to even loss of earnings in students’ future working life (Dorn et al., 2020b).

## Data Availability

The datasets used and/or analysed during the current study are available from the corresponding author on reasonable request.

## Appendix

### Appendix A Data extraction coding schema

**Table:**

Data type (Excel column headings)	Data codes to use within column cells
*Study characteristics*	
Sample N	Write in total number
Males N	Write in number of males
Females N	Write in number of females
Age	Write in mean age or range
Edu level	“K6” if from Grade 1 to grade 6; “K6-12”if from grade 6 to grade12; “K12” if from grade1 to grade 12-; “U” if were undergraduates; “G” if were graduates and post-graduates; “O” if others, or unclear
Subject	“STEM” if belongs to science, technology, engineer or mathematics; “Med” if relates nursing, anatomy, medicine etc “FD” if relates to food science, culinary etc “Edu” if belongs to general education “Psy.” if relates to psychology “Art” if related to arts subjects “Lang.” if belongs to language education “Econ.” if related to economics
Stakeholder	Write in types of stakeholders involved in study I.e., students/peers; parents/guardians; teachers; headmasters
*Academic cooperation*	
Single-authored	Coded as type “S”
Two authors within X institution, on a national level	Coded as type “TN” + write in number of institutions
Three and more authors within X institution, on a national level	Coded as type “MN” + write in number of institutions
Two authors within X institution, on an international level	Coded as type “TI” + write in number of institutions
Three and more authors within X institution, on an international level	Coded as type “MI” + write in number of institutions
*Conceptualization of student engagement*	Write in theory name + dimensions of engagement (i.e., behavioural; cognitive; emotional/affective; agentic; social)
*Methods*	
Types of data	“Q” = Questionnaire data; “I” = Interview data; “O” = Observation data; “T|= Trace data (includes task completion data; “P” = Physiological data; “OTH” = Other data
Approaches	“QUAN” if it’s quantitative; “QUAL” if it’s qualitative; “MX” if article applied mixed approaches
Instrument	If yes, write in the name of instrument; If not reported, mark as “NA”
Reliability of instrument (as measured by Cronbach’s alpha)	If yes, write in “α = ”; If not reported, mark as “NA”
*Strategies used to facilitate engagement*	Write in the specific strategies used in study
Others	
